# Diarrhea after pancreatic surgery is associated with the extent of resection: a single-center retrospective cohort-study

**DOI:** 10.1007/s00423-025-03936-w

**Published:** 2025-12-08

**Authors:** Charlotte Gustorff, Carl-Stephan Leonhardt, Jakob Mühlbacher, Tarek Hammoud Al-Darwisch, Mawe-Jakob Kirchrath, Klaus Sahora, Martin Schindl, Oliver Strobel, Ulla Klaiber

**Affiliations:** Department of General Surgery, Division of Visceral Surgery, Medical University of Vienna, Währinger Gürtel 18-20, Vienna, 1090 Austria

**Keywords:** Pancreatic surgery, Postoperative complications, Postoperative diarrhea, Neurogenic diarrhea

## Abstract

**Purpose:**

Diarrhea after pancreatic surgery is gaining growing importance since extended pancreatic resections have been increasingly performed. The aim of this study was to determine the incidence of diarrhea after pancreatic surgery with a special focus on the extent of resection and subgroups at higher risk for diarrhea.

**Methods:**

Retrospectively collected data of all consecutive patients undergoing pancreatic surgery between 01/2021 and 11/2023 were analyzed. Information on bowel movements was prospectively documented. Diarrhea was defined as > 3 bowel movements per day for at least 72 h despite pancreatic enzyme replacement and in the absence of laxatives or prokinetics. Extended resections were differentiated according to the type of vascular resection and arterial divestment. Clinicopathological characteristics and outcomes were compared among these groups and risk factors for diarrhea were identified.

**Results:**

A total of 320 patients were included. Following any type of pancreatectomy, 71/320 (22.2%) patients developed diarrhea. The incidence of diarrhea after partial pancreatoduodenectomy, distal pancreatectomy and total pancreatectomy was 26.6%, 11.5% and 35.3%, respectively (*p* = 0.004). Arterial divestment/resection and venous resection were significantly associated with an increased risk for postoperative diarrhea in 87% (OR = 31.14; 95%-CI: 8.77, 170.08; *p* < 0.001) and 52.2% of patients (OR = 5.14; 95%-CI: 2.51, 10.52; *p* < 0.001), respectively. Postoperative diarrhea was significantly associated with a prolonged length of hospital stay (19 vs. 13 days; 95%-CI: 3.00, 7.00; *p* < 0.001).

**Conclusion:**

Diarrhea after pancreatic resection is a common postoperative complication affecting especially patients undergoing extended resections with vascular resections and arterial divestment. Diarrhea significantly impairs postoperative recovery leading to a prolonged hospital stay.

## Introduction

 Postoperative digestive disorders are common following pancreatic resection [1]. Diarrhea after pancreatic resection can be caused by the loss of pancreatic exocrine tissue leading to pancreatic exocrine insufficiency [2], by removal of intestinal nerves with concomitant dysregulation of digestion leading to neurogenic diarrhea [3], as well as by bacterial overgrowth exacerbated by neoadjuvant chemotherapy and perioperative antimicrobial treatment [4]. Lacking an internationally accepted definition for diarrhea after pancreatic surgery, there is a wide span of reported incidences in available evidence as well as probably an underreporting of this clinically relevant adverse outcome. The incidence of diarrhea following pancreatic resection has been documented to range between 0 and 100% with great variance in definition, duration of follow-up as well as type of study and therewith level of evidence [5–8]. With the improvement of neoadjuvant treatment and refinements in surgical techniques, an increasing percentage of patients with borderline resectable and locally advanced pancreatic cancer (PDAC) can be subjected to surgical resection [9–11]. A recently published meta-analysis quantified resection rates after neoadjuvant treatment for borderline resectable and locally advanced PDAC with 60.6% and 22.2%, respectively [12]. Lymph node retrieval and resection margin status are key determinants for postoperative survival in PDAC independent from the treatment strategy [13–16]. Aiming at a considerable survival benefit, extended resections including vascular resections, TRIANGLE excision and arterial divestment are therefore frequently required to achieve complete tumor clearance [17, 18]. Especially, extended dissections/divestment along the celiac axis/hepatic artery and superior mesenteric artery (so-called level-3 dissection) [19] might cause refractory neurogenic diarrhea due to unavoidable injury of the regulating nerve plexus [3]. However, evidence on procedure-related incidence of diarrhea after pancreatic surgery is sparse.

The aim of this retrospective single-center cohort study was therefore to generate data on the incidence of diarrhea after several types of pancreatic resections and to identify risk factors associated with diarrhea during the postoperative course.

## Methods

### Study design and patient selection

This STROBE-compliant [20] single-center cohort study of consecutive patients undergoing formal pancreatic resection used the retrospective pancreatic database of the Division of Visceral Surgery, Department of General Surgery, Medical University of Vienna, Vienna, Austria. The primary objective was to determine the incidence of diarrhea after formal pancreatic resections and to identify risk factors for its occurrence. All indications warranting any type of formal pancreatic resection, i.e. partial pancreatoduodenectomy, distal or total pancreatectomy, were included. The study was approved by the institutional ethics committee on February 28, 2024 (approval no. 2127/2023). All consecutive patients undergoing formal pancreatic resection between January 1, 2021, and November 30, 2023, were included. Patients undergoing exploration without resection or parenchyma-sparing resection such as enucleation or central pancreatectomy were excluded. Resectability criteria were based on cross-sectional imaging findings at diagnosis and defined according to the consensus statement of the International Study Group of Pancreatic Surgery (ISGPS) [21]. Patients with stage IV tumors were also excluded except oligometastatic disease with complete regression of metastasis during chemotherapy. Parts of this work belong to diploma theses.

### Perioperative management

Neoadjuvant treatment was performed in all patients with locally advanced tumors at diagnosis. In the absence of standardized evidence for borderline-resectable situations [22], the treatment strategy was determined following multidisciplinary tumor board recommendations and individual patient’s as well as surgeon’s preference. Combination chemotherapy with FOLFIRINOX was the standard treatment, but Gemcitabine-based alternatives were considered at the oncologist’s discretion depending on patient’s performance status, age, comorbidities and treatment response after restaging. Notably, a considerable number of patients started (palliative) chemotherapy in external centers and were only referred for a second opinion.

Upfront surgery or surgical exploration following neoadjuvant treatment was undertaken once the serum carbohydrate antigen (CA) 19 − 9 levels had decreased considerably and if cross-sectional imaging excluded tumor progression. After exclusion of distant tumor spread, resection was performed using the uncinate-first and artery-first approaches depending on the individual tumor localization [23, 24]. Formal resections were carried out as partial pancreatoduodenectomy, distal or total pancreatectomy as indicated from an oncological perspective and as technically required. The pancreatic and distal bile duct transection margins as well as any suspicious tissue surrounding critical vascular structures were assessed during surgery by frozen-section microscopy. Depending on intraoperative findings extended procedures with venous and/or arterial resections were performed in selected cases [18, 25]. Arterial divestment was preferred over entire vessel resection whenever technically possible and oncologically reasonable [26]. The TRIANGLE operation and Inoue level-3 dissection were routinely performed in cancer patients to achieve radical clearance of lymphatic tissue between the superior mesenteric artery, celiac axis, portal vein/superior mesenteric vein and the mesopancreas [27, 28].

Postoperative procedures were carried out according to the institution’s evidence-based standard procedures. Usually, patients were observed for one day at the intensive-care unit (ICU) and then transferred to the normal ward on postoperative day 1. Perioperative thoracic epidural analgesia was routinely started intraoperatively and continued during the first postoperative days. Pancreatic enzyme replacement therapy (PERT) was administered according to the recommendations given by the (International Study Group of Pancreatic Surgery (ISGPS) [29] and the European guidelines for treatment of pancreatic exocrine insufficiency (PEI) [2]. Adjuvant treatment and post-resection completion chemotherapy were usually recommended under consideration of the extent of neoadjuvant treatment, the patient’s treatment tolerance, histopathologic tumor response, and in accordance with the institution’s multidisciplinary tumor board.

### Outcomes and follow-up

Patient clinical characteristics including the American Society of Anesthesiologists (ASA) score, comorbidities, and body mass index (BMI), disease-specific information including diagnosis and neoadjuvant treatment, as well as surgical information were extracted from the institution’s pancreatic database. The primary outcome of the study was postoperative diarrhea defined as more than 3 bowel movements per day for at least 72 h in the postoperative course despite adequate PERT and in the absence of laxatives or prokinetics. This definition was chosen based on definitions used in previous studies [30, 31] and under consideration of the postoperative specifics after pancreatic resection. Information about bowel movements was prospectively documented. The administration of PERT commenced postoperatively from the time point at which patients tolerated normal oral nutrition; this is routinely postoperative day 4. Following the recommendations by the ISGPS and the European guidelines for treatment of PEI, PERT started with 50,000 IE lipase per main meal and with 10–25,000 IE per snack after partial pancreatectomy [2, 29]. After total pancreatectomy PERT started with 75,000 IE lipase per main meal and with 25,000 IE per snack. Depending on clinical symptoms of PEI such as diarrhea or steatorrhea including high-volume and/or faddy stools as well as bloating, stepwise dose escalation was applied. In persistent and watery diarrhea, typically occurring directly after food intake, loperamide was started and tincture of opium was also added as required.

As second outcome parameters overall morbidity according to Clavien-Dindo [32], pancreatic surgery-associated complications (i.e., postoperative pancreatic fistula (POPF), delayed gastric emptying (DGE), postpancreatectomy hemorrhage (PPH), and chyle leak defined according to the ISGPS [33–36]), 90-day mortality, lengths of stay at hospital and intensive-care-unit, as well as reoperations and readmissions to hospital were assessed. Surgery- and disease-associated risk factors for diarrhea were identified. Patients were followed-up for 90 days after the index operation.

### Statistical analysis

For quantitative statistics, the calculation of continuous variables was performed as median values with interquartile ranges (IQR). Categorical variables were calculated as absolute and relative frequencies. A comparison between patients with and without diarrhea was tested employing the Wilcoxon-Mann-Whitney test for continuous variables and the Person’s Chi-squared test with Yates’ continuity correction for categorical variables. Fisher-test was used to calculate for Odds-Ratios (OR), the Hodges-Lehmann estimator for respective 95%-confidence intervals (CI). A multivariate logistic regression analysis was performed to identify factors associated with postoperative diarrhea as a binary outcome variable. OR were calculated with 95%-CI. Statistical significance was considered as a two-tailed p-value of < 0.05. Imputation was not performed because missing data was rare. All statistical analyses were conducted using R version 4.5.0 (The R Foundation for Statistical Computing) [37].

## Results

### Study cohort

A total of 320 patients received formal pancreatic resection during the study period between January 1, 2021, and November 30, 2023, and fulfilled eligibility criteria. See Table 1 for all baseline characteristics of the study cohort. The median age at the time of the operation was 70 years (IQR, 62.0 to 78.0 years) with an equal proportion of male and female patients (161 males and 159 females). Indications for surgery were predominantly PDAC in 148/320 (46.3%) patients, followed by intraductal papillary mucinous neoplasm (IPMN) in 56/320 (17.5%) patients, other malignant diseases in 48/320 (15.0%) patients and neuroendocrine tumors (NET) in 35/320 (10.9%) patients.

**Table 1:** Baseline characteristics

Parameter	*n* = 320
Age, years (IQR)	70.0 (62.0, 78.0)
Sex, male	161 (50.0%)
BMI, kg/m2 (IQR)	24.8 (22.6, 28.1)
Diagnosis	
PDAC	148 (46.3%)
IPMN	56 (17.5%)
NET	35 (10.9%)
Chronic pancreatitis	13 (4.1%)
Other malign	48 (15.0%)
Other benign	20 (6.3%)
ASA-Score	
I	10 (3.1%)
II	107 (33.4%)
III	197 (61.6%)
IV	6 (1.9%)
Initial resectability status	
Resectable	263 (82.2%)
Borderline resectable	50 (15.6%)
Unresectable	6 (1.9%)
Metastatic	1 (0.3%)
Neoadjuvant treatment FOLFIRINOX-based Gemcitabine-based Other regime No neoadjuvant treatment	35 (10.9%) 23 (65.7%) 11 (31.4%) 1 (2.9%) 285 (89.1%)
Comorbidities	
Diabetes mellitus	107 (33.4%)
Arterial hypertension	183 (57.2%)
Coronary artery disease	51 (15.9%)
Alcohol consumption	85 (26.6%)
Nicotine abuse	113 (35.3%)
Type of pancreatic resection	
Partial pancreatoduodenectomy	199 (62.2%)
Distal pancreatectomy	104 (32.5%)
Total pancreatectomy	17 (5.3%)
Arterial divestment	23 (7.2%)
Arterial resection	5 (1.6%)
Venous resection	46 (14.4%)

*Data are medians with interquartile ranges (IQR) or numbers of patients with percentages. PDAC = pancreatic ductal adenocarcinoma*,* IPMN = intraductal papillary mucinous neoplasia*,* NET = neuroendocrine tumor*,* ASA = American Society of Anesthesiologists physical status score.*

Two-hundred and sixty-three of 320 (82.2%) patients were diagnosed with primarily resectable stage of disease at diagnosis. Borderline resectability was initially diagnosed in 50/320 (15.6%) patients, while local unresectability was found in 6/320 (1.9%) patients and oligometastatic disease at diagnosis in 1/320 (0.3%) patient with complete regression of metastasis during neoadjuvant treatment. 35 out of 320 (10.9%) patients received neoadjuvant treatment with a FOLFIRINOX-protocol in most patients (23/35 patients, 65.7%), while approximately one third of patients received a gemcitabine-based regime (11/35 patients, 31.4%). The majority of patients scored for an American Society of Anesthesiologists (ASA) physical status score of III (197/320 patients, 61.6%). Most prevalent comorbidities were arterial hypertension in 183/320 (57.2%) patients, diabetes mellitus in 107/320 (33.4%) patients, and coronary artery disease in 51/320 (15.9%) patients.

All patients included underwent formal pancreatic resection. Partial pancreatoduodenectomy was performed in 199/320 (62.2%) patients, distal pancreatectomy in 104/320 (32.5%) patients and total pancreatectomy in 17/320 (5.3%) patients. Partial and total pancreatoduodenectomies were performed open in all patients. Distal pancreatectomies were performed with an open approach in 76, laparoscopic in 26 and robotic in 2 patients. Venous resection, i.e. resection of the porto-mesenteric axis with concomitant reconstruction was performed in 46/320 (14.4%) patients, while arterial resection, i.e. resection and reconstruction of the superior mesenteric artery, hepatic artery or the celiac trunk was carried out in 5/320 (1.6%) patients. Arterial divestment was performed in 23/320 (7.2%) patients.

### Postoperative outcomes

Postoperative surgical results are summarized in Table 2. Major postoperative morbidity (Clavien-Dindo grades ≥ 3) occurred in 84/320 (26.3%) of patients. Postoperative 90-day mortality was 3.1%. POPF grades B/C affected 85/320 (26.6%) patients. PPH grades B/C occurred in 18/320 (5.6%) patients. DGE grades B/C were observed in 44/320 (13.8%) patients. Chyle leak was diagnosed in 42/320 (13.1%) patients. Reoperation was required in 29/320 (9.1%) cases. Fifty of 320 (15.6%) patients were readmitted to a surgical ward during the 90-day follow-up period. There was no readmission due to diarrhea.

**Table 2:** Postoperative outcomes

Parameter	*n* = 320
Overall morbidity, Clavien-Dindo grades ≥ 3	84 (26.3%)
90-day mortality	10 (3.1%)
Reoperation	29 (9.1%)
Readmission to hospital	50 (15.6%)
POPF	
Biochemical leak	66 (20.6%)
POPF B	70 (21.9%)
POPF C	15 (4.7%)
PPH	
ISGPS type A	7 (2.2%)
ISGPS type B	12 (3.8%)
ISGPS type C	6 (1.9%)
DGE	
ISGPS type A	24 (7.5%)
ISGPS type B	30 (9.4%)
ISGPS type C	14 (4.4%)
Chyle leak ISGPS type A	42 (13.1%)
Length of hospital stay Median (IQR), *days*	14 (10.0; 21.0)
Length of ICU-stay, Median (IQR), *days*	1 (1.0; 2.0)

*Data are medians with interquartile ranges (IQR) or numbers of patients with percentages. DGE = delayed gastric emptying*,* POPF = postoperative pancreatic fistula*,* PPH = postpancreatectomy hemorrhage*,* ISGPS = International Study Group of Pancreatic Surgery*,* ICU = intensive care unit*

**Table 3:** Univariate analysis of potential risk factors for postoperative diarrhea

	Total	Diarrhea	No Diarrhea	Statistics
All patients	320	71 (22.2%)	249 (77.8%)	
Diagnosis PDAC IPMN NET	148 56 35	43 (29.1%) 6 (10.7%) 4 (11.4%)	105 (70.9%) 50 (89.3%) 31 (88.6%)	χ²=6.55, *p* = 0.010, OR = 2.07 (95%-CI: 1.17; 3.71). φ = 0.14^Ω^ χ²= 4.34, *p* = 0.037, OR = 0.37 (95%-CI: 0.12; 0.92). φ = 0.12 ^Ω^ χ²=2.09, *p* = 0.149, OR = 0.41 (95%-CI: 0.10; 0.23). φ = 0.08 ^Ω^
Dignity				
Malignant disease	194	56 (28.9%)	138 (71.1%)	χ²=10.87, *p* < 0.001, OR = 2.89 (95%-CI: 1.51; 5.81;), φ = 0.28 ^Ω^
Non-malignant disease	126	15 (11.9%)	111 (88.1%)	
Type of pancreatic operation				
Partial pancreatoduodenectomy	199	53 (26.6%)	146 (73.4%)	χ²=5.36, p = **0.021**, OR = 2.07 (95%-CI: 1.11; 3.98), φ = 0.13 ^Ω^
Distal pancreatectomy	104	12 (11.5%)	92 (88.5)	χ²=9.22, *p* = 0.002, OR = 0.35 (95%-CI: 0.16; 0.69), φ = 0.17 ^Ω^
Total pancreatectomy	17	6 (35.3%)	11 (64.7)	χ² =1.06, *p* = 0.3, OR = 1.99 (95%-CI: 0.58; 6.15), φ = 0.06 ^Ω^
Extent of resection				
Arterial divestment/arterial resection	23	20 (87.0%)	3 (13.0%)	χ²=55.29, *p* < 0.001, OR = 31.14 (95%-CI: 8.77; 170.08), φ = 0.42 ^Ω^
Venous resection	46	24 (52.2%)	22 (47.8%)	χ²= 25.31, *p* < 0.001, OR = 5.14 (95%-CI: 2.51; 10.52), φ = 0.28 ^Ω^
Standard	261	40 (15.3%)	221 (84.7%)	χ² = 41.59, *p* < 0.001, OR = 0.14 (95%-CI: 0.07; 0.27), φ = 0.08 ^Ω^
Neoadjuvant treatment	35	19 (54.3%)	16 (45.7%)	χ²=20.73, *p* < 0.001, OR = 5.21 (95%-CI: 2.34; 11.56), φ = 0.26 ^Ω^
Length of hospital stay, Median (IQR), *days*	14 (10.0; 21.0)	19 (13.0; 29.0)	13 (10.0; 19.0)	*p* < 0.001 (95%-CI: 3.00; 7.00)^Σ^
Length of ICU-stay, Median (IQR), *days*	1 (1.0; 2.0)	1 (1.0; 2.0)	1 (1.0; 2.0)	*p* = 0.121 (95%-CI: −1.00; 2.85) ^Σ^

*Data are medians with interquartile ranges (IQR) or numbers of patients with percentages. OR = Odds-ratio*,* CI = confidence interval*,* Ω = Pearson’s Chi-squared test with Yates’ continuity correction; fisher-test for Odds-Ratio; Σ = Wilcoxon-Mann-Whitney-Test*,* Hodges-Lehmann estimator for confidence intervals. PDAC = pancreatic ductal adenocarcinoma. IPMN = intraductal papillary mucinous neoplasm*,* NET = neuroendocrine tumor*,* ICU = intensive care unit.*

### Risk factors for postoperative diarrhea

In patients with non-malignant disease, diarrhea was detected with a significantly lower but still clinically relevant incidence with 15/126 (11.9%) patients being affected. In patients with IPMN, the incidence of postoperative diarrhea was as high as 10.7% (6/56 patients with IPMN), while 4/35 (11.4%) patients with NET developed postoperative diarrhea.

Arterial resection or arterial divestment led to postoperative diarrhea in 20/23 patients and this was associated with a significantly increased risk of developing diarrhea with an observed incidence of 87% (OR = 31.14; 95%-CI: 8.77, 170.08; *p* < 0.001). Further, among patients undergoing venous resection 42/24 (52.2%) patients developed postoperative diarrhea leading to a significant increase in risk for postoperative diarrhea (OR = 5.14; 95%-CI: 2.51, 10.52; *p* < 0.001). Multivariate analysis confirmed arterial resection or arterial divestment to be a statistically significant risk factor for postoperative diarrhea with an OR of 11.35 (95%-CI: 2.01, 91.86; *p* = 0.010), while venous resections were not an independent risk factor for postoperative diarrhea (see Table 4).

**Table 4:** Multivariate analysis of potential risk factors for postoperative diarrhea

Variable	OR	95% CI		*p*-value
PDAC	0.99	0.443	2.341	0.996
IPMN	0.71	0.205	2.297	0.576
Malignant disease	1.51	0.521	4.497	0.449
Partial pancreatoduodenectomy	0.45	0.148	1.469	0.165
Distal pancreatectomy	0.25	0.073	0.898	**0.028**
Venous resection	0.34	0.043	1.838	0.240
Arterial divestment/arterial resection	11.35	2.007	91.858	**0.010**
Neoadjuvant treatment	1.46	0.542	3.785	0.446
Length of hospital stay	1.036	1.007	1.047	**0.008**

*OR Odds ratio*,* CI confidence interval*,* PDAC pancreatic ductal adenocarcinoma*,

*IPMN intraductal papillary mucinous neoplasm.*

Neoadjuvant treatment significantly impacted the risk of developing postoperative diarrhea in univariate analysis (OR = 5.21, 95% CI 2.34–11.56; *p* < 0.001). In multivariate analysis, neoadjuvant treatment was not a significant risk factor. Incidence of diarrhea among patients undergoing neoadjuvant treatment was 54.3% (19/35 patients).

The median length of hospital days was 14 days (IQR, 10 to 21). Patients with diarrhea had a significantly prolonged hospital stay with a median of 19 days (IQR, 13.0 to 29.0; 95%-CI: 3.0, 7.0; *p* < 0.001). In multivariate analysis, length of hospital stay was significantly associated with postoperative diarrhea (OR = 1.04; 95%-CI: 1.01, 1.05; *p* = 0.008). Median length of ICU-stay was 1 day (IQR, 1.0 to 2.0) and showed no statistically significant association with postoperative diarrhea (*p* = 0.120).

## Discussion

This single-center retrospective cohort study including 320 patients undergoing formal pancreatic resection, shows that diarrhea after pancreatic surgery is a frequent clinical finding occurring in 22.2% of all patients. Patients suffering from malignant disease and especially PDAC had a significantly increased risk for developing postoperative diarrhea compared to patients with non-malignant entities in univariate but not in multivariate analysis. However, diarrhea was a clinically relevant complication in all subgroups of patients. Extended surgery with arterial resection or at least arterial divestment increased the risk for postoperative diarrhea significantly and independently from confounding variables. Venous resection was not confirmed being an independent risk factor which might be explained by less radical surgery in tumors with venous involvement only.

This is one of the first studies focusing on postoperative diarrhea after pancreatic resection. Even though diarrhea has been frequently reported in former studies as secondary outcome parameter, reported incidences are inconclusive as definitions of diarrhea are inconsistent or even frequently lacking [30, 38]. Boggi and co-workers reported that all patients after pancreatectomy with arterial resection suffered from manageable diarrhea [38]. However, a clear definition of diarrhea was not given. Klotz et al. analyzed the incidence of diarrhea defined as >3 bowel movements per day and “regular use of anti-diarrheal medication in postoperative week two” in patients undergoing partial or total pancreatoduodenectomy with or without TRIANGLE procedure. Incidences of postoperative diarrhea were 14.4% versus 34.4% after partial pancreatoduodenectomy without versus with TRIANGLE, respectively. After total pancreatectomy, incidence of diarrhea was reported to be 29.2% in both groups (with and without TRIANGLE) [30]. In line with this, more extended resections, like partial pancreatoduodenectomy and total pancreatectomy, lead to increased incidences of postoperative diarrhea in our cohort. The incidence of diarrhea in patients undergoing total pancreatectomy was highest compared to other procedures. Nevertheless, the increase in the risk of diarrhea after total pancreatectomy was not statistically significant. This might be due to the small number of total pancreatectomies included in the study. It should be noted that the majority of patients undergoing arterial resection did not lose the entire pancreas to save venous gastric drainage while at the same time pancreatic anastomosis is usually at low risk in patients with locally advanced PDAC after extensive neoadjuvant treatment.

Patients undergoing neoadjuvant treatment had significantly increased risk for diarrhea as shown in the univariate analysis, with 54% of patients being affected in this study. This can be explained by the fact that patients undergoing neoadjuvant treatment all had advanced cancer (mostly PDAC) requiring not only preoperative chemotherapy but also extended surgery with Inoue level-III resections or at least TRIANGLE combined with arterial divestment and/or vascular resections. However, neoadjuvant treatment was not a statistically significant risk factor for diarrhea in multivariate analysis which could point towards neoadjuvant treatment being a confounding factor since neoadjuvant treated patients might need more extended resections and might therefore be more prone to diarrhea. The results from this study are in line with previously published data from China, reporting a high incidence of 44% of postoperative diarrhea in patients with PDAC undergoing neoadjuvant treatment [39]. Further, diarrhea is one of the most common adverse events of neoadjuvant treatment with FOLFIRINOX reported in 11% of patients with borderline-resectable PDAC in a recent meta-analysis [40].

Arterial divestment and vascular resections have been demonstrated to result in a substantial and significant increase in the risk of developing diarrhea in the present study. This supports the concept of neurogenic diarrhea caused by resection of the periarterial nerve plexus surrounding the celiac trunk and superior mesenteric artery. To our experience neurogenic diarrhea has an impact on clinical management because naturally this cannot be managed by PERT but needs extensive anti-diarrheal medication comprising not only a combination of anti-propulsive drugs such as loperamide at maximum dosage and tincture of opium but also anti-secretory drugs such as enkephalinase inhibitors. Typically, neurogenic diarrhea is characterized by low-volume watery stools occurring immediately after meals with a high frequency per day depending on oral food intake. This contrasts with diarrhea caused by PEI which is typically characterized by high-volume non-watery stools. Of course, PEI must be ruled out and effectively prevented after pancreatic resection. The European guidelines for the diagnosis and treatment of PEI recommend an initial dose of 40,000–50,000 units of lipase with main meals, followed by 20,000–25,000 units with snacks [2]. After pancreatic surgery, especially total pancreatectomy, higher dosages of PERT are frequently required as recommended by the ISGPS and these should be stepwise increased depending on the individual patient’s symptoms [2, 29].

In this study, diarrhea after surgery significantly prolonged the length of hospital stay for a median of 6 additional days indicating the clinical relevance of postoperative diarrhea. Depending on the severity of diarrhea in terms of daily stool frequency and duration of diarrhea, it can be assumed that it can considerably hamper patients’ postoperative recovery and weight gain or even lead to weight loss. Comparable to the results by Napoli et al. [38] postoperative diarrhea was manageable with oral medication including PERT, loperamide, tincture of opium and enkephalinase inhibitors in this study. Diarrhea did not lead to organ failures such as renal insufficiency requiring intensive care treatment. In general, patients in this cohort study recovered quickly with a median postoperative ICU-stay of one day mostly for monitoring reasons. Longer ICU-stays in individual cases can be referred to other postoperative complications rather than diarrhea. The prolonged hospitalization in patients with diarrhea could in parts also be explained by other factors such as increased morbidity in the subgroup of patients with extended surgery, but also prolonged surveillance just due to the increased risk for severe complications in patients undergoing arterial resection.

A previous study by the Dutch Pancreatic Cancer Group showed diarrhea to negatively impact quality of life three years after pancreatic surgery [41]. The current retrospective study only focused on diarrhea during the first 90 days after surgery. Thus, data on the longer postoperative course are needed to investigate the long-term consequences of diarrhea, especially after extended resections with intestinal denervation. Napoli et al. reported that diarrhea predominantly characterizes the first 6 months after surgery which was the follow-up period of their study [38].

This study has several limitations mainly based on the retrospective single-center character of the study design bearing risk of bias. On the other hand, diarrhea was prospectively documented in this study, but daily documentation was limited to the duration of hospitalization. Further, factors such as antibiotic treatment and dietary changes such as fat-free diet were not analyzed and could influence bowel motility.

In addition, the study cohort is quite heterogenous including any indications for pancreatic resection and therewith several types of pancreatic resections. However, the intention was to assess postoperative diarrhea after formal pancreatic resections for different indications including PDAC as well as premalignant and benign indications necessitating less radical resection. Even though malignant disease was associated with diarrhea in univariate analysis, this was not confirmed in multivariate analysis, suggesting that the association is indirect and driven by the need for more extensive surgery rather than malignancy itself. The limited sample size might also be an explanation.

A widely accepted definition for diarrhea after pancreatic surgery is necessary to make inter-study comparisons possible and such a definition is currently elaborated by the ISGPS.

In conclusion, this is one of the first studies focusing on diarrhea after pancreatic resection and the results show that diarrhea is a frequent and clinically relevant complication resulting in prolonged hospital stay. Due to neurogenic denervation PDAC patients with advanced stages undergoing conversion surgery with extended resection following neoadjuvant treatment are at increased risk for developing diarrhea in the postoperative course. Further data on the severity of neurogenic diarrhea and management are required.

## Data Availability

No datasets were generated or analysed during the current study.

## References

[1] Zhang C, Zironda A, Vierkant RA et al (2024) Quality of life and gastrointestinal symptoms in long-term survivors of pancreatic cancer following pancreatoduodenectomy. Ann Surg 279(5):842–849 37497660 10.1097/SLA.0000000000006053

[2] Dominguez-Muñoz JE, Vujasinovic M, de la Iglesia D et al (2025) European guidelines for the diagnosis and treatment of pancreatic exocrine insufficiency: UEG, EPC, EDS, ESPEN, ESPGHAN, ESDO, and ESPCG evidence-based recommendations. United Eur Gastroenterol J 13(1):125–172 10.1002/ueg2.12674 PMC11866322 39639485

[3] Kuroki N, Ono Y, Sato T et al (2022) Long-term outcome of patients with postoperative refractory diarrhea after tailored nerve plexus dissection around the major visceral arteries during pancreatoduodenectomy for pancreatic cancer. World J Surg 46(5):1172–1182 35119513 10.1007/s00268-022-06457-5

[4] Bures J, Cyrany J, Kohoutova D et al (2010) Small intestinal bacterial overgrowth syndrome. World J Gastroenterol 16(24):2978–2990 20572300 10.3748/wjg.v16.i24.2978 PMC2890937

[5] Suzuki S, Miura J, Shimizu K et al (2016) Clinicophysiological outcomes after total pancreatectomy. Scand J Gastroenterol 51(12):1526–1531 27461044 10.1080/00365521.2016.1211173

[6] Dresler CM, Fortner JG, McDermott K et al (1991) Metabolic consequences of (regional) total pancreatectomy. Ann Surg 214(2):131–140 1867520 10.1097/00000658-199108000-00007 PMC1358512

[7] Kawai M, Tani M, Hirono S et al (2014) Pylorus-resecting pancreaticoduodenectomy offers long-term outcomes similar to those of pylorus-preserving pancreaticoduodenectomy: results of a prospective study. World J Surg 38(6):1476–1483 24370543 10.1007/s00268-013-2420-z

[8] Wang ML, Shyr BS, Chen SC et al (2023) Comparison of robotic and open central pancreatectomy. Int J Med Robot e2562 10.1002/rcs.2562 37574857

[9] van Veldhuisen E, Klompmaker S, Janssen QP et al (2023) Surgical and oncological outcomes after preoperative FOLFIRINOX chemotherapy in resected pancreatic cancer: an international multicenter cohort study. Ann Surg Oncol 30(3):1463–1473 36539580 10.1245/s10434-022-12387-2 PMC9908650

[10] Wijetunga AR, Chua TC, Nahm CB et al (2021) Survival in borderline resectable and locally advanced pancreatic cancer is determined by the duration and response of neoadjuvant therapy. Eur J Surg Oncol 47(10):2543–2550 33952409 10.1016/j.ejso.2021.04.005

[11] Strobel O, Neoptolemos J, Jäger D et al (2019) Optimizing the outcomes of pancreatic cancer surgery. Nat Rev Clin Oncol 16(1):11–26 30341417 10.1038/s41571-018-0112-1

[12] Brown ZJ, Heh V, Labiner HE et al (2022) Surgical resection rates after neoadjuvant therapy for localized pancreatic ductal adenocarcinoma: meta-analysis. Br J Surg 110(1):34–42 36346716 10.1093/bjs/znac354

[13] Goess R, Jäger C, Perinel J et al (2024) Lymph node examination and survival in resected pancreatic ductal adenocarcinoma: retrospective study. BJS Open. 10.1093/bjsopen/zrad125 38271272 10.1093/bjsopen/zrad125 PMC10810280

[14] Leonhardt CS, Hank T, Pils D et al (2024) Prognostic impact of resection margin status on survival after neoadjuvant treatment for pancreatic cancer: systematic review and meta-analysis. Int J Surg 110(1):453–463 38315795 10.1097/JS9.0000000000000792 PMC10793837

[15] Strobel O, Hank T, Hinz U et al (2017) Pancreatic cancer surgery: the new R-status counts. Ann Surg 265(3):565–573 27918310 10.1097/SLA.0000000000001731

[16] Strobel O, Hinz U, Gluth A et al (2015) Pancreatic adenocarcinoma: number of positive nodes allows to distinguish several N categories. Ann Surg 261(5):961–969 24979603 10.1097/SLA.0000000000000814

[17] Diener MK, Mihaljevic AL, Strobel O et al (2021) Periarterial divestment in pancreatic cancer surgery. Surgery 169(5):1019–1025 33032819 10.1016/j.surg.2020.08.030

[18] Loos M, Kester T, Klaiber U et al (2022) Arterial resection in pancreatic cancer surgery: effective after a learning curve. Ann Surg 275(4):759–768 33055587 10.1097/SLA.0000000000004054

[19] Inoue Y, Saiura A, Takahashi Y (2018) A novel classification and staged approach for dissection along the celiac and hepatic artery during pancreaticoduodenectomy. World J Surg 42(9):2963–2967 29464344 10.1007/s00268-018-4550-9

[20] von Elm E, Altman DG, Egger M et al (2007) The strengthening the reporting of observational studies in epidemiology (STROBE) statement: guidelines for reporting observational studies. PLoS Med 4(10):e296 17941714 10.1371/journal.pmed.0040296 PMC2020495

[21] Bockhorn M, Uzunoglu FG, Adham M et al (2014) Borderline resectable pancreatic cancer: a consensus statement by the international study group of pancreatic surgery (ISGPS). Surgery 155(6):977–988 24856119 10.1016/j.surg.2014.02.001

[22] Klaiber U, Leonhardt CS, Strobel O et al (2018) Neoadjuvant and adjuvant chemotherapy in pancreatic cancer. Langenbecks Arch Surg 403(8):917–932 30397779 10.1007/s00423-018-1724-8

[23] Hackert T, Werner J, Weitz J et al (2010) Uncinate process first–a novel approach for pancreatic head resection. Langenbecks Arch Surg 395(8):1161–1164 20582600 10.1007/s00423-010-0663-9

[24] Müller PC, Müller BP, Hackert T (2023) Contemporary artery-first approaches in pancreatoduodenectomy. Br J Surg 110(12):1570–1573 37327072 10.1093/bjs/znad175

[25] Hartwig W, Gluth A, Hinz U et al (2016) Outcomes after extended pancreatectomy in patients with borderline resectable and locally advanced pancreatic cancer. Br J Surg 103(12):1683–1694 27686238 10.1002/bjs.10221

[26] Schneider M, Hackert T, Strobel O et al (2021) Technical advances in surgery for pancreatic cancer. Br J Surg 108(7):777–785 34046668 10.1093/bjs/znab133

[27] Hackert T, Strobel O, Michalski CW et al (2017) The TRIANGLE operation - radical surgery after neoadjuvant treatment for advanced pancreatic cancer: a single arm observational study. HPB (Oxford) 19(11):1001–1007 28838632 10.1016/j.hpb.2017.07.007

[28] Inoue Y, Saiura A, Oba A et al (2019) Optimal extent of superior mesenteric artery dissection during pancreaticoduodenectomy for pancreatic cancer: balancing surgical and oncological safety. J Gastrointest Surg 23(7):1373–1383 30306451 10.1007/s11605-018-3995-3

[29] Gianotti L, Besselink MG, Sandini M et al (2018) Nutritional support and therapy in pancreatic surgery: a position paper of the international study group on pancreatic surgery (ISGPS). Surgery 164(5):1035–1048 30029989 10.1016/j.surg.2018.05.040

[30] Klotz R, Hackert T, Heger P et al (2022) The TRIANGLE operation for pancreatic head and body cancers: early postoperative outcomes. HPB (Oxford) 24(3):332–341 34294523 10.1016/j.hpb.2021.06.432

[31] Klaiber U, Alldinger I, Probst P et al (2016) Duodenum-preserving pancreatic head resection: 10-year follow-up of a randomized controlled trial comparing the beger procedure with the Berne modification. Surgery 160(1):127–135 27106794 10.1016/j.surg.2016.02.028

[32] Dindo D, Demartines N, Clavien PA (2004) Classification of surgical complications: a new proposal with evaluation in a cohort of 6336 patients and results of a survey. Ann Surg 240(2):205–213 15273542 10.1097/01.sla.0000133083.54934.ae PMC1360123

[33] Bassi C, Marchegiani G, Dervenis C et al (2017) The 2016 update of the international study group (ISGPS) definition and grading of postoperative pancreatic fistula: 11 years after. Surgery 161(3):584–591 28040257 10.1016/j.surg.2016.11.014

[34] Wente MN, Veit JA, Bassi C et al (2007) Postpancreatectomy hemorrhage (PPH): an International Study Group of Pancreatic Surgery (ISGPS) definition. Surgery 142(1):20–25 17629996 10.1016/j.surg.2007.02.001

[35] Wente MN, Bassi C, Dervenis C et al (2007) Delayed gastric emptying (DGE) after pancreatic surgery: a suggested definition by the International Study Group of Pancreatic Surgery (ISGPS). Surgery 142(5):761–768 17981197 10.1016/j.surg.2007.05.005

[36] Besselink MG, van Rijssen LB, Bassi C et al (2017) Definition and classification of chyle leak after pancreatic operation: a consensus statement by the international study group on pancreatic surgery. Surgery 161(2):365–372 27692778 10.1016/j.surg.2016.06.058

[37] R: A language and environment for statistical computing (Version 4.5.0). Version 4.5.0 ed.: R Foundation for Statistical Computing; 2025:Software

[38] Napoli N, Kauffmann EF, Lombardo C et al (2024) Postoperative results, learning curve, and outcomes of pancreatectomy with arterial resection: a single-center retrospective cohort study on 236 procedures. Int J Surg 110(10):6111–6125 38079592 10.1097/JS9.0000000000000971 PMC11486960

[39] Zhu J, Wang Y, Li Y et al (2024) Risk factors of post-operative diarrhoea in patients with pancreatic cancer after neoadjuvant chemotherapy: a retrospective cohort study. J Clin Nurs 33(5):1777–1785 38426618 10.1111/jocn.17040

[40] Janssen QP, Buettner S, Suker M et al (2019) Neoadjuvant FOLFIRINOX in patients with borderline resectable pancreatic cancer: a systematic review and patient-level meta-analysis. J Natl Cancer Inst 111(8):782–794 31086963 10.1093/jnci/djz073 PMC6695305

[41] Latenstein AEJ, Blonk L, Tjahjadi NS et al (2021) Long-term quality of life and exocrine and endocrine insufficiency after pancreatic surgery: a multicenter, cross-sectional study. HPB (Oxford) 23(11):1722–1731 34001452 10.1016/j.hpb.2021.04.012

